# Frequency and circumstances of placebo use in clinical practice - a systematic review of empirical studies

**DOI:** 10.1186/1741-7015-8-15

**Published:** 2010-02-23

**Authors:** Margrit Fässler, Karin Meissner, Antonius Schneider, Klaus Linde

**Affiliations:** 1 Institute of Biomedical Ethics, University of Zurich, 8008 Zurich, Switzerland; 2 Institute of Medical Psychology, Ludwig-Maximilians University, 80336 Munich, Germany; 3 Institute for General Practice, Technische Universität München, 81667 Munich, Germany

## Abstract

**Background:**

The use of placebo interventions outside clinical trials is ethically, professionally and legally controversial. Little is known about the frequency and circumstances of placebo use in clinical practice. Our aim was to summarize the available empirical studies addressing these issues.

**Methods:**

We searched PubMed and EMBASE from inception to July 2009 in order to identify cross-sectional surveys, qualitative or longitudinal studies among health care professionals, students or patients which investigated at least one of the following issues - frequency of placebo use or attitudes to, or motivations for, the use of placebo interventions. At least two reviewers extracted information on the study methods, participants and findings. Descriptive summaries were prepared in an iterative process by at least two reviewers per study.

**Results:**

Twenty-two studies from 12 different countries met the inclusion criteria. Most studies had relevant shortcomings. The proportion of respondents reporting that they had applied 'pure' placebos (for example, saline injection) during their professional life varied between 17% and 80% among physicians and between 51% and 100% among nurses, but it seems that the actual frequency of such use seems to be rare. The use of 'impure' or 'active' placebos (for example, antibiotics for viral infections) is likely to be much more frequent. However, it is impossible to make a reliable estimation because there is no agreement of what an impure placebo might be. Studies using qualitative methods or asking participants to judge case examples suggest that motivations and attitudes towards placebo use are complex and health care providers are often faced with a dilemma.

**Conclusions:**

Although the available evidence is incomplete and confusing at times there can be little doubt that the prevalence of placebo use outside of clinical trials is not negligible and that views and attitudes on placebos use differ considerably among individuals, both health care professionals and patients. Further research is needed to clarify these issues.

## Background

Placebos are crucially important for the evaluation of health care interventions but they are also used in clinical practice. There are many definitions of placebos [1] and the overall concept is problematic [2-4]. However, in general, placebos are thought to be interventions which do not contain components that will improve the condition being treated. Inert interventions such as 'sugar pills' or saline injections are often designated 'pure' placebos, whereas therapies that contain active components, but are considered ineffective for the condition being treated, are called 'impure' placebos (for example, antibiotics in viral infections) [5]. The use of placebo interventions in clinical practice is ethically, professionally and legally controversial [6-8] and it is important that the frequency and the circumstances in which placebos are used outside clinical trials should be investigated. Our aim was to systematically summarize the available empirical data on: the use of placebos in clinical practice; the respective motivations; and the attitudes of health care professionals, students and patients towards their use.

## Methods

### Data sources and searches

The basic requirements of the literature search - inclusion criteria, selection process and extraction - were specified in advance in a short protocol which can be obtained from the authors. Potentially relevant articles were searched in PubMed (from 1966 to July 2009) and EMBASE (from 1974 to July 2009) and by citation tracking. We identified relevant studies using combinations of words used in the titles (for example, "medical practice", "placebo", "placebo therapy", "survey", "use of placebo") and/or controlled terms (such as, "attitude", "attitude of health personnel, health knowledge, attitudes", "practice", "patient care", "interview", "medical practice", "physician-patient relations", "placebos", "placebo effect", "questionnaires").

### Selection criteria

In order to be included articles had to meet the following criteria: (1) they had to be original reports of cross-sectional surveys, qualitative studies or longitudinal studies; (2) participants had to be health care professionals, students, or patients; and (3) studies had to investigate at least one of the following issues: frequency of the use of placebo interventions, attitudes to or motivations for the use of placebo interventions outside the setting of clinical trials. We did not have *a priori *restrictions regarding language or publication type, but our search only identified full-length articles in English and German. One reviewer (MF) screened all the hits of the electronic search and excluded those which were clearly irrelevant. All reviewers checked their own extensive files of papers on placebo and the reference lists of included studies for further potentially relevant publications. All those papers which were considered potentially relevant were read in full text by three reviewers and independently assessed for eligibility. Initial disagreements occurred on three studies which were resolved by discussion.

### Data extraction, data synthesis and quality assessment

Due to the great variability of included studies, the extraction and summarizing of relevant information was done in an iterative process. As a first step three reviewers independently extracted the basic information regarding: the topics addressed; the methodological approach; sampling; response rates; participants; whether or not the original questions were available; the definitions of placebos provided; and whether pure and impure placebos were separated or whether there was a focus on one or both of these types of placebos. In the second step at least two reviewers independently extracted the results. Thirdly, based on these extractions, one reviewer compiled tables which summarized the findings of each study regarding the frequency of placebo use, conditions or reasons of placebo use, use of placebo for separating organic and functional diseases and personality issues, beliefs in the effectiveness and ethical issues. Study findings which could not be summarized reasonably in the table format were summarized separately. The summary tables and the separate summaries were read by two other reviewers and potential improvements to the format were discussed. In the fourth step, a second reviewer re-extracted all trials in the same table format. The fifth step involved the comparison of the summaries and the final version was established.

As quality indicators for questionnaire-based quantitative surveys at least two reviewers assessed the sampling method and documented the response rate. Samples were classified as 'convenience' (for example, questionnaires distributed at a conference or in a hospital), 'local' (more systematic full or random samples based on local structures) or 'random' (random samples of clearly defined national or larger regional populations). For the qualitative studies, we documented whether systematic sampling, data collection or analytical methods were used and described.

## Results

### General overview and methodological quality of included studies

**Figure 1:**
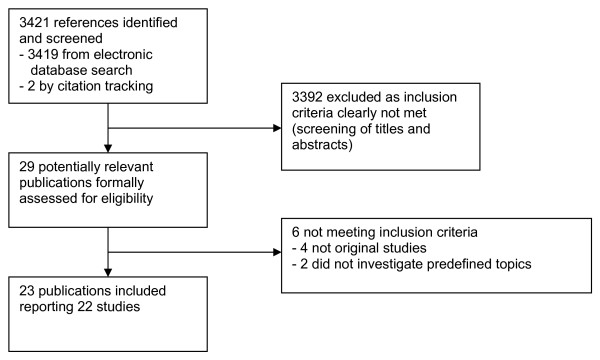
**Study flow diagram**.

The literature search identified a total of 3421 references (see Figure 1). Twenty-nine publications were formally assessed for eligibility. Four were not original studies [9-12] and two only addressed issues that were indirectly related to placebo [13,14]. A total of 22 studies published in 23 articles between 1973 and 2009 [15-37] met the inclusion criteria (see Table 1 and Additional File 1). Twenty were quantitative surveys in which a total of 29 samples of individuals were asked to answer questionnaires (19 studies), or were interviewed using a highly structured questionnaire (one study [29], and subgroups of participants in two [20,22]). Sixteen samples comprised physicians, nine comprised nurses, three comprised patients and one study included medical interns or medical students. Most surveys had important shortcomings. We classified three samples as random, six as local and 20 as convenience samples. Response rates ranged from 48% to 65% among random samples, from 36% to 94% among local and from 48% to 100% among convenience samples (seven studies using convenience sampling did not report on response rates). Two of the studies reporting questionnaire-based surveys additionally included prospective substudies in which records of hospital patients were regularly screened for placebo use for 6 months [19] or a year [21]. Two publications reported qualitative studies in which physicians had been interviewed [17,25]. Both described sampling methods but only one [25] provided details on data collection and analysis methods.

**Table 1:** Overview of included studies.

First author year	from	*N*	Participants	Sampling	Response rate	Focus pure/impure placebo
**Questionnaire-based quantitative surveys**						
Shapiro 1973 [15,16]	USA	195	Ph (various groups)	Convenience	83%	Both
Goldberg 1979 [18]	USA	102	N (head nurses at 11 hospitals)	Local	68%	Pure
Goodwin 1979 [19]	USA	60	Ph (house officers)	Convenience	100%	Mainly
		39	N (hospital nurses)	Convenience	Unclear	pure
		27	License practical nurses/medical aides	Convenience	Unclear	
Gray 1981 [20]	CAN	70	Ph (university hospital)	Convenience	82%*	Pure
		230	N (university hospital)	Convenience		
		35	N (experienced hospital nurses)	Convenience	Unclear	
Lange 1981 [21]	GER	81	Ph, N, psychologists (no data for subgroups provided)	Convenience	Unclear	Pure
Thomson 1982 [22]	NZ	37	Ph (GPs)	Local	84%	Both
Classen 1985 [23]	GER	101	Ph (setting unclear)	Convenience	Unclear	Both
Saupe 1986 [24]	GER	56	N (at a psychiatric university hospital)	Convenience	80%	Mainly pure
Lynöe 1993 [26]	SWE	94	Ph (GPs or affiliated with university)	Local	94%	Mainly
		83	Pt (consecutive patients of three GPs)	Local	83%	impure
Ernst 1997 [27]	AUS	263	N (setting unclear)	Convenience	58%	Both
Berger 1999 [28]	USA	74	Medical interns at an university-affiliated hospital	Convenience	83%	Pure
Berthelot 2001 [29]	FRA	300	Pt (at a hospital rheumatology department)	Convenience	Unclear	Mainly
		100	N (same hospital, various departments)	Convenience	Unclear	pure
Hrobjartsson 2003 [30]	DEN	502	Ph (GPs, hospital, specialists in private practice)	Random	65%	Both
Nitzan 2005 [31]	ISR	31	Ph (senior hospital physicians)	Convenience	76%	Mainly
		31	N (head nurses from same hospitals)	Convenience	100%	pure
		27	Ph (family physicians)	Convenience	68%	
Lim 2007 [32]	SIN	402	Medical students	Local	36%	Pure
Sherman 2007 [33]	USA	231	Ph (faculty members of 3 medical schools)	Local	50%	Both
Tilburt 2008 [34]	USA	679	Ph (internists and rheumatologists)	Random	57%	Both
Bernateck 2009 [35]	GER	71	Ph (university hospital)	Convenience	80%*	Pure
		107	N (university hospital)	Convenience		
Chen 2009 [36]	NZ	211	Pt (in waiting rooms of two GP clinics)	Convenience	48%	Mainly pure
Fässler 2009 [37]	SWI	233	Ph (primary care)	Random	48%	Both
**Substudies with prospective screening of medical records of hospital patients**						
Goodwin 1979 [19]	USA	1900	Pt (academic teaching hospital, treated during 6 months)	n.a.		Pure
Lange 1981 [21]	GER	1725	Pt (psychiatric hospital, all treated 1978)	n.a.		Pure
**Qualitative studies**						
Comaroff 1976 [17]	UK	47	Ph (GPs)	Local	92%	Mainly impure
Schwartz 1989 [25]	USA	72	Ph (selected for often prescribing inefficient drugs)	n.a.	51%	Impure

*Response rate only reported for pooled groups.

Ph = physicians; N = nurses; Pt = patients; GPs = general practitioners; n.a. = not applicable.

### Definitions of placebo

Definitions of placebo in the studies varied considerably (Additional File 2). Six studies did not address definitional issues in the publication and probably in the questionnaire but five of these clearly focused on pure placebos [18-21,31], while some of the questions in one survey also addressed impure placebos [27]. Nine studies included explicit definitions or explanations in questionnaires or interviews for clarification [23,28-30,32,34-37]. One of these explicitly asked participants not to proceed with the questionnaire if they disagreed with the definition [30]. Three studies made the definitional aspects a topic of the questionnaire. Fässler *et al*. [37] had provided definitions for pure and impure placebos but gave participants the option to rate examples provided as '...is not a placebo intervention'. This option was chosen by 2% to 32% of the respondents. Sherman *et al*. [33] asked participants to choose between several definitions (see Additional File 2 for responses). Shapiro [15,16] listed 21 examples of interventions and a formal definition and asked participants to rate the degree of their agreement on whether these interventions should be considered placebos. Apart from four examples related to inert drugs, the agreement was less than 70% for all other listed interventions (see Additional File 2 for examples). The remaining four studies [17,22,25,26] addressed their topic indirectly (with case vignettes and resulting behaviour or through addressing non-scientific drug prescribing) and bypassed the definitional issues.

### Frequency of placebo use

The proportion of physicians who reported having used placebo (ever used or used with a minimum frequency of once a year) varied between: 17% and 80% for pure placebos (six studies); between 54% and 57% for impure placebos (two studies); and between 41% and 99% if both pure and impure placebos were addressed (five groups of physicians in three studies; see Table 2). The pure placebos mentioned were typically saline injections, sugar pills or prepared placebo tablets. Typical impure placebos mentioned included antibiotics for viral infections, vitamins and analgesics for unproven indications. However, in studies offering these options, more than half of respondents also reported the use of non-essential diagnostic interventions [37] and not specifically indicated physiotherapy [30]. The use of impure placebos seems to be more prevalent in primary care settings; pure placebos seem to be used more frequently in hospital settings. The reported frequency of placebo was less than once a month in the majority of the studies which reported this information but findings were highly variable depending on the placebo definition used and the sampling frame. The proportion of hospital nurses reporting use of (mainly pure) placebos (51% to 100% in eight studies) was higher than in surveys of physicians, but the actual frequency of placebo applications was low. The only study of interns found that 16% had witnessed the application of a pure placebo; 2% (1 of 47) had actually given a placebo. In the two studies, based on the review of the medical records of hospital inpatients, the incidence of the application of pure placebos (mostly single applications) was 0.3% during 6 months (various departments in a USA hospital) [19] and 5.1% during 12 months (a psychiatric hospital in Germany) [21].

**Table 2:** Summary of findings on the frequency of placebo use.

Author year	Setting	Type	Placebo use	Definition of use	Frequency or other additional information
**Questionnaire- based quantitative surveys**					
*Physicians*					
Goodwin 1979 [19]	Hospital (house officers)	Pure*	78%	Ever use for pain relief	
Gray 1981 [20]	Hospital	Pure	80%	Ever use	
Classen 1985 [23]	Unclear	Pure	60%	Use 'sometimes'	About 30% less than once a month
Classen 1985 [23]	Unclear	Impure	54%	Use 'sometimes'	
Lynöe 1993 [26]	Unclear	Both	99%	Ever use	None very often, 1% often, 4% rather often, 26% quite rarely, 68% rarely/very rarely
Hrobjartsson 2003 [30]	General practice	Both	86%	During the last year	38% 1-10 times/year, 48% > 10 times/year
Hrobjartsson 2003 [30]	Hospital	Both	54%	During the last year	44% 1-10 times/year, 10% > 10 times/year
Hrobjartsson 2003 [30]	Specialist practice	Both	41%	During the last year	31% 1-10 times/year, 10% > 10 times/year
Nitzan 2004 [31]	Hospital/family practice	Pure*	53%	Use with a minimum frequency of once a year	37% once a month or more often†
Sherman 2007 [33]	Medical school faculty	Both	45%	Ever use	15% 1-10×, 8% > 10×, 22% not at all during last year
Tilburt 2008 [34]	Specialist practice	Both	80%	Ever use	34% = once a month, 28% 2-3 times/month, 18% = once a week
Bernateck 2009 [35]	Hospital	Pure	52%	Use with a minimum frequency of once a year	40% 1-2 times/year, 9% 1-2 times/month, 4% more often
Fässler 2009 [37]	Primary care	Pure	17%	Ever use	93% once a month or less often
Fässler 2009 [37]	Primary care	Impure	57%	Ever use	
*Nurses*					
Goldberg 1979 [18]	Hospital	Pure	51%	Ever use	44% with current use or use in the last 6 months
Goodwin 1979 [19]	Hospital	Pure*	82%	Ever use for pain relief	
Gray 1981 (sample 1) [20]	Hospital	Pure	80%	Ever use	
Gray 1981 (sample 2) [20]	Hospital	Pure	89%	At least once during the last 5 years	63% during the last year
Saupe 1985 [24]	Hospital	Pure	100%	Ever use	79% once or less per month, 21% more often
Ernst 1997 [27]	Unclear	Pure	57%	Ever use	
Ernst 1997 [27]	Unclear	Both	68%	Ever use	12% during the last year
Nitzan 2004 [31]	Hospital	Pure*	71%	Use with a minimum frequency of once a year	37% once a month or more often†
Bernateck 2009 [35]	Hospital	Pure	87%	Use with a minimum frequency of once a year	45% 1-2 times/year, 33% 1-2 times/month, 9% more often
*Interns*					
Berger 1999 [28]	Hospital	Pure	16%	Ever witnessed use	Only 2% (1/47) had actually given a placebo
**Substudies with prospective screening of medical records of hospital patients**					
Goodwin 1979 [19]	Hospital	Pure*	0.3%	During 6 months (prospective)	
Lange 1981 [21]	Psychiatric hospital	Pure	5.1%	During 12 months (prospective)	6.1% in women, 3.9% in men

* Limitation to pure placebo not explicitly stated but questions/report with clear focus on pure placebo use.

† Numbers related to both physicians and nurses.

### Indications and reasons for placebo use, placebo personality and beliefs in placebo effectiveness

The conditions for which placebos were used and the reasons for use were not clearly separated in some studies. Therefore, they are summarized together (Table 3). Typical indications reported for the use of pure placebos, particularly in hospital settings, were pain, insomnia, anxiety and risk of substance abuse. A consistent finding was that patients who were considered as more difficult or demanding were more likely to receive a pure placebo. In primary care settings physicians reported the desire of patients to receive a prescription as a primary motivation for the use of (particularly, impure) placebos. Other reasons often reported were to take advantage of the placebo effect, to avoid conflicts with patients, as supplemental treatment or for non-specific symptoms and to avoid telling patients that treatment possibilities were exhausted. The proportion of physicians and nurses having used (pure) placebos for diagnostic purposes or who believed that it is possible to differentiate organic and functional disease varied greatly between studies (between 4% and 70%; see Additional File 3). More recent studies reported lower rates (between 4% and 29%) and nurses tended to report higher rates than physicians. Seven studies included one or more questions on whether a variety of factors might predict placebo response (Additional File 3). Findings suggest that the majority of physicians, nurses and patients believe that personality elements are associated with the likelihood of a patient responding to placebo. Physicians tended to think that other physicians, or other specialities than their own, make more use of placebo (six studies). Questions regarding beliefs in the effectiveness of placebo applications varied greatly (Additional File 4). Up to 50% of physicians and nurses (range 16% to 50%) believed that placebo treatments are either always, often, or generally effective. Similarly, the perceived percentage of patients responding to placebo treatments ranged between 5% and 42%. In the prospective study of psychiatric patients 45% of the placebo administrations were rated as successful [21]. Between 25% and 33% of doctors and medical students believed that placebo treatments can induce not only subjective/psychological, but also objective or physiological changes. The two surveys in patients addressing the issue [29,36] suggest that patients are slightly more sceptical about placebo effectiveness than physicians and nurses.

**Table 3:** Summary of findings regarding conditions in which placebos are used and/or reasons for using placebos.

First author year	Indications/reasons
Goldberg 1979 [18]	N: In 39 of 43 (91%) patient's pain was an indication. For 36 patients more than one reason was reported; in 51% anxiety; in 47% symptoms suspected not to be organic; in 33% in suspected drug abuse; in 24% as nothing else was helping; in 16% fear of iatrogenic addiction; in 7% concern for patient safety; in 37 of 43 patients receiving placebo (86%) anxiety and emotional problems were seen as prominent; 28% were considered less likeable; 30% more difficult than average; 81% were considered questionable or unreliable.
Goodwin 1999 [19]	Ph/N: 87% of physicians and 97% of registered nurses who had used a placebo in the past had ordered it for a patient requiring more pain medication that thought necessary; 74% and 84% had ordered it in 'problem patients'
Gray 1981 [20]	Ph/N: Conditions: 89% pain (45% patients with regular narcotic analgesia; 34% postoperative; 8% terminally ill); 9% anxiety. More than half of replies indicated that patients describing features such as manipulative, complaining, and histrionic behaviour usually received placebos.
Lange 1981 [21]	Diagnoses in the 88 patients receiving placebo from the 1725 psychiatric patients surveyed: 27% schizophrenia and paranoid symptoms; 27% abuse; 19% depressive psychoses; 14% hysteric syndromes. Symptoms treated with placebo: 40% pain; 29% sleep problems; 8% agitation. Reasons for placebo application: most frequently to cope with a difficult situation and in frequently complaining or disliked patients. Further reasons: other interventions not successful; avoid substance abuse; patients desire for receiving drug treatment.
Classen 1985 [23]	Ph: In patients who demand too many, too powerful or inadequate drugs (50%); psychosomatic complaints (42%); pain (36%); somatic disorders (7%).
Saupe1986 [24]	N: 72% in demanding patients; 78% in patients with so called psychosomatic complaints; 67% requests for pain relief.
Ernst 1997 [27]	N: Probably open-ended question (responses given by placebo users): 19% pain; 11% insomnia; 7% both; 8% anxiety; 5% addiction.
Berger 1999 [28]	Medical interns: Likely to use a placebo in the following circumstances: Suspicion of factitious pain (48%); history of substance abuse (18%); psychiatric illness/psychological component (17%).
Hrobjartsson 2003 [30]	Ph: 226 (45%) respondents provided examples: 90 used various placebos as for treating pain; 86 antibiotics for viral infections; 32 vitamins against fatigue; 28 various placebos for cough and chronic obstructive lung disease. Reported reasons (total sample): follow the wish of the patient and avoid conflicts with patients (70% GPs, 46% hospital clinicians, 42% private specialists); take advantage of the placebo effect (48%, 22%, 32%); avoid discontinuation of other prescriptions (40%, 27%, 18%); avoid telling patients that treatment possibilities are exhausted (36%, 11%, 17%).
Nitzan 2004 [31]	Ph/N: Conditions: The medical conditions for which the placebos were used included anxiety, pain (including abdominal), agitation, vertigo, sleep problems, asthma, contractions in labour, withdrawal from recreational drugs, and angina pectoris (when the blood pressure was too low to allow for vasodilators). Reasons: 43% after 'unjustified' demand of medication; 38% to calm the patient; 38% as analgesic; 28% as diagnostic tool; 23% as adjunctive therapy; 17% for non-specific complaints; 15% to buy time before next regular dosage of medication; 11% to get patient to stop complaining.
Sherman 2007 [33]	Ph: Among placebo users 18% used placebos to calm the patient; 18% as supplemental treatment; 15% after 'unjustified' demand for medication; 13% for non-specific symptoms; 11% after all treatment possibility were exhausted; 6% to control pain; 6% to get the patient stop complaining.
Bernateck 2009 [35]	Ph: Conditions (responses given by placebo users): 76% (65% physicians, 81% nurses) for pain; 59% insomnia (40%, 66%); 12% depressive mood (19%, 10%). Reasons for placebo application: 64% (57%, 66%) patient's request for a drug; 37% (35%, 38%) for calming an anxious patient; 35% (24%, 40%) for reducing drug use; 20% (30%, 16%) for supporting other interventions; 18% (24%, 16%) to treat non-specific symptoms.
Fässler 2009 [37]	Ph: 69% of placebo users report as motive 'to gain therapeutic advantage through the placebo effect'; 64% 'to offer a treatment to patients whose complaints and test results are not attributable to a certain disease'; 63% 'to conform with the requests of the patient'; 51% 'to offer treatment to difficult patients'; 44% 'to offer a treatment option to a patient with an incurable disease'; 37% 'in situations in which standard treatments may burden patients with side effects or are contraindicated'; 31% 'to avoid drug addiction'.

Ph = physicians; N = nurses; GP = general practitioner.

### Ethical aspects

Only a small minority of the physicians and nurses participating in the included studies thought that the use of placebos should be categorically prohibited or considered it should never be permissible (see Additional File 5). At the same time the findings show that the majority considered the use of placebos as problematic. For example, in one study 73% reported that placebo use means deceiving the patient [27] and, in another survey, 45% agreed to the statement that placebo use must be rejected as it implies deception [37]. A minority of physicians seems to have no, or only a few, reservations to the use of placebo. The surveys of patients indicate that opinions on whether placebo application is appropriate are highly divergent and strongly dependent on the specific situation [26,29,38]. If placebos are applied for the benefit of the patient, up to 50% or even more consider it acceptable.

### Narrative summary of studies not fitting into the extraction format

Three studies did not fit into our formal extraction scheme and are summarized narratively. In the study by Lynöe *et al*. [26] 83 patients and 94 physicians were given the same questionnaire which described three hypothetical cases and then asked to judge the situations and actions (see Additional File 6). The results showed that judgements about the acceptability of a defined action depended on specific situations and varied strongly both between and within the patients group and the physicians group. In general, there was tension between the physicians' respect for the patients' wishes and the patients' respect for the physician's professional autonomy. For example, 65% of patients and 30% of physicians agreed that physicians ought to oblige a patient's desire even if they consider a treatment to be a placebo, while 60% and 87%, respectively, agreed that patients ought to respect a physician's refusal to give a treatment she/he considers to be a placebo.

In a qualitative study addressing placebo use indirectly Comaroff [17] interviewed 47 general practitioners (GPs) in Wales. She first asked physicians to estimate the proportion of consultations that culminated in a prescription. In the majority of cases this induced a discussion on the adequacy of high prescription rates and only partly on the placebo effect. The emerging pattern of responses showed that GPs shared the strong implicit professional ideal that treatment should always be specific and prescribed only when necessary. However, this ideal conflicted with the uncertainty and ambiguity in many situations in general practice. One of the options to prevent a possibly unsettling hesitation was the use of therapies which, according to scientific theory, are not or not fully indicated. The GPs justified this inadequate use - according to professional standards - by stating that they were satisfying the patient's expectations. Other important justifications were the beneficial effects associated with any therapy or with some sort of faith in the specific effects of the therapy despite conflicting external evidence. Placebo therapy or unscientific prescribing also seemed to serve a need to give the patient the feeling of being cared for in spite of the lack of time that GPs were allowed to give at any one appointment.

Schwartz *et al*. [25] interviewed, in a standardized manner, 72 physicians who had been identified from a Medicaid database as prescribing drugs of doubtful effectiveness more often than average. Similar to the results described by Comaroff [17], they found that the most frequent justification (51 from a total of 110 reasons provided) was patient demand, often combined with the fear of losing patients. A distinct subgroup of physicians considered their positive personal experience with the drugs prescribed as being more important and valid than scientific studies (26% of statements). In 24% of responses the placebo effect was reported as a reason.

## Discussion

### Summary of main findings

The results of the available surveys show that a significant proportion of physicians and nurses have applied 'pure' placebos (such as saline injection or sugar pills) during their professional life, but the actual frequency of such use seems to be rare except for a small minority of frequent users. The use of 'impure' or 'active' placebos (for example, antibiotics for viral infections) is likely to be much more frequent but, due to the lack of agreement on what actually has to be considered an impure placebo and to the unclear influence of the social desirability of placebo, when health care professionals are asked to admit its use it is not always possible to produce reliable estimations. In particular, the studies using qualitative methods, or asking participants to judge case vignettes, show that motivations and attitudes towards placebo use are complex, and often cause a dilemma for health care providers. In this situation quantitative surveys might miss many relevant issues.

### Strengths and limitations

To the best of our knowledge this is the first systematic review which summarizes the available empirical data on the actual use of placebos in clinical practice and the related attitudes and beliefs. As our predefined selection criteria were wide, our study set is highly heterogeneous regarding the design, samples, quality and topics addressed. We consider it to be a strength of our review that it provides the overall picture. This shows that the available evidence is disparate, which points to the enormous complexity of the issue. We were fascinated by the multiple facets of the placebo problem detected in our study sample exactly *because *of its heterogeneity. However, for the process of a systematic review such heterogeneity is associated with drawbacks. The methodological quality of the included studies could only be assessed in a basic manner. Nevertheless, it is obvious that most studies had relevant shortcomings. For example, the reported frequencies of placebo use might be biased by the use of convenience sampling or low response rates - or both. Our attempt to cover a broad variety of aspects made it difficult to summarize the results of primary studies in a transparent manner. Therefore, we provide detailed tables that allow the reader to check our narrative summaries and to see which numbers refer to which study. Due to the methodological shortcomings and the heterogeneity of the primary studies there is room for interpretation.

### Interpretation

There is broad consensus that interventions such as saline injections for pain or sugar pills are (pure) placebos. The application of such pure placebos can be characterized relatively easily: the provider is aware and convinced that he is applying something which has no direct, specific or physiological effect. The application is almost always deceptive, in the sense that the patient is not informed of the provider's view and, if fully informed, the patient also would probably consider the intervention to be a placebo. Pure placebos are mainly used as single or short-term applications and, typically, in situations which are difficult for some reason. Many of the reasons for applying pure placebos seem to be ethically and professionally difficult, such as the use in patients who are demanding too much medication. However, there are also many situations where the compromise between helping and deceiving the patient is much more difficult. The advantage of pure placebos compared to impure placebos is that there is no direct toxicity (although nocebo effects - negative effects associated with the application of an inert intervention - can occur [39]). Therefore, doing no harm is an often reported reason in cases when pure placebos are used. There seems to be a small minority of physicians who make frequent use of pure placebos. It would seem worthwhile to investigate the motivations of these physicians. The available data suggests that, in hospitals, nurses tend to use (pure) placebos more frequently than physicians. This finding could be due to the fact that each nurse receives orders from several physicians. A further explanation could be that nurses more often have to deal with difficult situations when caring for patients the care or that the closer nurse-patient interaction in many hospitals accounts for this difference. The few available surveys of patients suggest that placebo application seems to be acceptable to many in certain situations.

Interpreting the available data on the use of impure placebos is difficult. What is considered to be an impure placebo varies considerably among studies and it is unclear and subjective when an intervention is a placebo or an active or effective intervention. Surveys investigating definitional aspects reveal considerable disagreement regarding whether defined interventions should be considered (impure) placebos or not [16,37]. Context and motivations for using impure placebos seem to be variable and often different than for pure placebos. With this lack of clarity it is doubtful whether the evaluation of something such as 'the prevalence of the use of impure placebos' makes sense. Figures might depend more on how questions are posed than on what participants actually think. For example, some of the authors of responses http://www.bmj.com/cgi/eletters/337/oct23_2/a1938 to the recent study by Tilburt *et al*. [34] believe that the comparably high rates of 'placebo' use in this survey are due to the specific wording of the relevant question which may result in physicians classifying interventions as placebo treatment when they normally would not consider them to be placebos. In qualitative studies addressing the prescription of antibiotics for sore throats physicians tend to acknowledge that they are well aware that their behaviour is problematic but the word placebo does not seem to come up [38,40]. The academic concept of an impure placebo might inappropriately reflect the complex situations and motivations in which health care professionals apply interventions which are not backed up by scientific evidence.

## Conclusions

Although there is a plethora of review articles and an increasing number of laboratory and clinical studies dealing with placebo and placebo effects, empirical investigations on the current use of placebos in clinical practice and on the respective attitudes of health care professionals, students or patients are sparse. While the available evidence is incomplete and partly confusing there can be little doubt that (a) the prevalence of the use of 'pure' and 'impure' placebos (or scientifically inadequate interventions) outside clinical trials is not negligible, and that (b) views and attitudes on placebos use differ considerably among individuals, both among health care professionals and patients. Future more carefully planned, rigorous, questionnaire-based quantitative surveys could provide additional information on placebo use and attitudes. Researchers should consider not only pilot testing questionnaires among a limited number of persons but also systematically interviewing participants of the pilot study to check whether questions have been really understood in the intended manner. Qualitative studies investigating in more detail the circumstances and motivations for placebo use or inadequate prescribing would be of particular interest. Based on the available evidence, we addressed the placebo issue almost exclusively from the perspectives of scientists and health care providers. Future studies should also investigate the perspectives of patients.

The authors believe that the use of pure placebos should be restricted to exceptional situations due to the deceptive element involved. In some uncertain situations health care professionals will probably (have to) continue to use interventions which are considered by scientists to be impure placebos. Physicians should be aware that there is a tendency to use problematic rationales (perceived patient expectations of prescriptions, unjustified faith in efficacy, using a prescription as a substitute of time) as legitimization and to repress the dilemmas associated with uncertainty. Relevant research on placebo might yield findings that could contribute to the development of a more honest and efficient patient-provider relationship.

## Abbreviations

GP: general practitioner.

## Competing interests

The authors declare that they have no competing interests.

## Authors' contributions

MF, KM and KL together conceptualized the review, selected and extracted the studies and performed the analysis process. MF did the main literature search. AS contributed to the interpretation. KL drafted the manuscript. All authors contributed to revising the manuscript and approved the final version. MF, KM and KL all acted as guarantors.

## Pre-publication history

The pre-publication history for this paper can be accessed here:

http://www.biomedcentral.com/1741-7015/8/15/prepub

## Supplementary Material

Additional file 1 Summary of findings regarding ethical issues. Click here for file

Additional file 2 Topics addressed in the included studies. Click here for file

Additional file 3 Definitions or aspects related to the definition of placebo/placebo effects in the included studies. Click here for file

Additional file 4 Summary of findings regarding beliefs on whether placebos can be used for diagnostic purposes (D) and personality features of responders (P). Click here for file

Additional file 5 Summary of findings regarding beliefs and experiences on effectiveness of placebo treatment. Click here for file

Additional file 6 **Summary of the study by Lynöe *et al***. [26]. Click here for file
