# Supplementary material for: Frequency and circumstances of placebo use in clinical practice - a systematic review of empirical studies
Source: BMC Med. 2010 Feb 23;8:15. doi: 10.1186/1741-7015-8-15 (PMC2837612; doi:10.1186/1741-7015-8-15)
Supplement: Additional file 1 — Summary of findings regarding ethical issues. [file 1741-7015-8-15-S1.DOC]

**Additional file 1: Topics addressed in the included studies**

| **First author year** | **Original questions available** | **Frequency of use** | **Types of placebos used** | **Indications/ reasons** | **Diagnostic use** | **Responder personality** | **Placebo use by other physicians** | **Beliefs in effectiveness** | **Ethical aspects** | **Major other issues** |
| --- | --- | --- | --- | --- | --- | --- | --- | --- | --- | --- |
| **Questionnaire-based quantitative surveys** | | | | |  |  |  |  |  |  |
| Shapiro 1973 [15,16] | Yes | (X) |  |  |  | X | X |  | X |  |
| Goldberg 1979 [18] |  | X |  | X | X |  |  | X | X |  |
| Goodwin 1979 [19] |  | X† |  | X† | X |  |  | X |  |  |
| Gray 1981 [20] |  | X | X‡ | X | (X) | X |  | X | X |  |
| Lange 1981 [21] | Yes* |  |  | X† | X | X |  | X† | (X) |  |
| Thomson 1982 [22] | In large part | (X) |  |  | X |  | X |  | (X) |  |
| Classen 1985 [23] | Yes* | X |  | X | X |  |  |  | X | 3 |
| Saupe 1986 [24] | Partly | X |  | X | X |  |  |  | X |  |
| Lynöe 1993 [26] | In large part | X |  |  |  |  | X |  | X |  |
| Ernst 1997 [27] |  | X | X | X | X | X |  | X | X |  |
| Berger 1999 [28] |  | X | X | X | X |  |  | X | X |  |
| Berthelot 2001 [29] | Yes |  |  | (X) |  | X |  | X | X | 2 |
| Hrobjartsson 2003 [30] | Yes* | X | X | X | X |  | X^*^ | X | X |  |
| Nitzan 2004 [31] | Yes | X | X | X | X |  |  | X | X | 1 |
| Lim 2007 [32] | Yes |  |  |  |  |  |  | X | X |  |
| Sherman 2007 [33] | Yes* | X | X | X | X |  | X | X | X | 1,6 |
| Tilburt 2008 [34] | Yes* | X | X |  |  |  |  |  | X | 1 |
| Bernateck 2009 [35] | Yes* | X |  | X | X |  |  | X |  | 1 |
| Chen 2009 [36] | Yes |  |  |  | X | X |  | X | (X) |  |
| Fässler 2009 [37] | Yes* | X | X | X | X | X |  | X | X | 1,5,6 |
| **Substudies with prospective screening of medical records of hospital patients** | | | | | |  |  |  |  |  |
| Goodwin 19791 [9] |  | X† |  | X† |  |  |  |  |  |  |
| Lange 1981 [21] |  | X | X | X† |  |  |  | X† |  |  |
| **Questionnaire-based quantitative surveys** | | | | |  |  |  |  |  |  |
| Comaroff 1976 [17] |  |  | (X) | X |  |  | (X) |  | (X) | 4 |
| Schwartz 1989 [25] |  |  |  | X |  |  |  |  | (X) | 6 |
| Sum of studies addressing question | | 15 | 10 | 15 | 13 | 5 | 6 | 13 | 17 |  |

Parentheses indicate that the topic had been addressed only indirectly; * = provided by authors; †ssues covered in both sub-studies of one publication were counted only once for the sum of studies addressing the question; ‡ = included also use in clinical trials

Major other issues: 1 = information provided when giving placebos; 2 = 59% of patients knew about the placebo effect; questions on adverse effects, reimbursement, and extent to which placebo effects contribute to various therapies ; 3 = factors influencing placebo effects: instruction sheet (75%), colour (60%), price (47%), form (40%), mode of application (39%), taste (32%); younger physicians and hospital physicians more likely to use pure placebos, hospital physicians also more likely to use impure placebos; 4 = focus on deeper reasons why physicians use unscientific prescribing and why they have major problems with placebos; 5 = potential reaction of patients if they become aware of placebo application; 6 = definition of placebo
