# Supplementary material for: Frequency and circumstances of placebo use in clinical practice - a systematic review of empirical studies
Source: BMC Med. 2010 Feb 23;8:15. doi: 10.1186/1741-7015-8-15 (PMC2837612; doi:10.1186/1741-7015-8-15)
Supplement: Additional file 2 — Topics addressed in the included studies. [file 1741-7015-8-15-S2.DOC]

**Additional file 2: Definitions or aspects related to the definition of placebo/placebo effects in the included studies**

| **First author year** | **Definition** |
| --- | --- |
| Shapiro 1973 [15,16] | Included 22 items where physicians were asked whether they agree (quantified using a score) that a given intervention was a placebo: 95% agreement for inert drug, 68% inert mechanical treatment, 54% for inert surgery; 66% for an active drug without specific activity for the condition treated, 50% for an active mechanical and 44% with an active surgical treatment; 47% treatment by quacks, 44% religious healers, 41% psychological treatment, 37% psychotherapeutic treatment and 36% psychoanalytic treatment; 58% agreement to the classical placebo definition by Shapiro |
| Classen 1985 [23] | Characterization using examples: “pure placebos (e.g. glucose, NaCl)”, “impure placebos, i.e. substances … judged as pharmacologically ineffective (e.g. homeopathic medicines, geriatrics)” |
| Berger 1999 [28] | “A placebo was defined as an inactive substance administered to a patient in place of a medication” (probably reported in the questionnaire) |
| Berthelot 2001 [29] | “…the placebo effect is an improvement produced when a compound devoid of pharmacological activity is given to patients who think it may be an active drug” |
| Hrobjartsson 2003 [30] | “Placebo treatment is defined in this survey as an intervention, without a ‘specific effect’ on a given state, but with a possible ‘unspecific effect’.” Additional information on original questionnaire: “Pure placebo treatment is treatment with interventions which aren't accepted to have a specific effect on a particular disease; e.g. treatment with calcium tablets. Impure placebo treatment is treatment with interventions which have a specific therapeutic effect on a certain condition, but not on current condition to be treated, e.g. treatment with penicillin in the case of a viral infection.” 7% disagreed with the definition and did not participate further |
| Lim 2007 [32] | “A substance containing no medication and prescribed or given to reinforce a patient’s expectation to get well, or something of no intrinsic remedial value that is used to appease or reassure another” |
| Sherman 2007 [33] | “51% of respondents endorsed the definition that a placebo is ‘an intervention that is not expected to have an effect through a known physiologic mechanism,’ 37% of the respondents chose ‘an intervention not considered to have a ‘specific’ effect on the condition treated, but with a possible ‘unspecific’ effect’, 28% of the respondents chose ‘an intervention that is inert or innocuous’, while 6% expressed other definitions…” |
| Tilburt 2008 [34] | Problems of definitions discussed in the introduction of the article; in the questionnaire: “placebo treatment” means “a treatment whose benefits derived from positive patient expectations and not from the physiologic mechanism of the treatment itself” |
| Bernateck 2009 [35] | Explanation in questionnaire: “With ‘placebo’ we mean here tablets without a specifically active substance or injections without specifically active substance” |
| Chen 2009 [36] | Placebo was described as an “inactive treatment such as a sugar pill that looks like the real pill and is given in the same way as a real pill.” However, without mentioning the word (active) placebo some questions also addressed active placebos |
| Fässler 2009 [37] | “Pure placebos are inert substances or methods such as sugar pills or isotonic saline solution. Impure placebos refer to substances or methods which have a known pharmacological or physical activity but which cannot be expected to have any direct therapeutic effects for the respective disease and in the chosen dosage, e.g. vitamin infusions for cancer or peppermint pills for pharyngitis.” Physicians had the possibility to state whether they did not consider the following examples of placebo interventions as placebo: positive suggestions (32%), simple ointments and/or bandages for contusions (27%), sugar pills (2%), saline injections (2%), therapies without pharmacological or physical activity in the condition (6%), non-essential physical examinations (19%), non-essential technical examinations (12%) |
