# Supplementary material for: Frequency and circumstances of placebo use in clinical practice - a systematic review of empirical studies
Source: BMC Med. 2010 Feb 23;8:15. doi: 10.1186/1741-7015-8-15 (PMC2837612; doi:10.1186/1741-7015-8-15)
Supplement: Additional file 3 — Definitions or aspects related to the definition of placebo/placebo effects in the included studies. [file 1741-7015-8-15-S3.DOC]

**Additional file 3: Summary of findings regarding beliefs on whether placebos can be used for diagnostic purposes (D), and regarding personality features of responders (P)**

| **First author year** | **Separating organic vs. functional problems/personality** |
| --- | --- |
| Shapiro 1973 [15,16] | P: Physicians were asked to rate agreement on a scale from 1 (completely agree) to 7 (completely disagree): placebo reactors have less intelligence 2.95 |
| Goldberg 1979 [18] | D: In 22% of the patients treated with placebo by nurses it was for diagnostic purpose; 14% of nurses believed that placebo use is acceptable for diagnostic purposes |
| Goodwin 1979 [19] | D: Among placebo users 60% (physicians) and 97% (nurses) had ordered placebo to determine whether pain reported by a patient was real  P: Depending on the specific case vignette given as example response to placebo was interpreted differently: case 1 – anxious, complaining patient 53% (physicians), 72% (registered nurses), 78% (medical aides); case 2 – crushing chest pain with shortness of breath 10% and 30% (no data for medical aides); clear trend to use placebos in “problem patients” |
| Gray 1981 [20] | D: 61% of nurses and physicians thought that the same relief of pain with a placebo as with morphine did not mean that the pain was not real, 22% replied that the patient was not experiencing “real pain”  P: 69% disagreed to the statement that patients with a major psychiatric diagnosis respond better to placebo than those without, 31% agreed. 22% thought the pain of a patient responding to placebo was not real |
| Lange 1981 [21] | Questionnaire-based survey - D: 66% of nurses and 20% of physicians/psychologists agreed to the statement “if a placebo does not help, the symptoms are real”, 63% vs. 33% to “if the placebo helps, the symptoms are imaginary”. Experienced nurses believed more strongly in the utility of placebos as a diagnostic tool  Screening of medical records - D: 3% of all placebo applications for diagnostic purposes (setting psychiatric hospital) |
| Thomson 1982 [22] | D: 19% (physicians) would use a placebo to find out whether a pain is organic or not, further 27% would do so in rare occasions |
| Classen 1985 [23] | D: 70% of the physicians administering placebos used them to test whether a symptom is real |
| Saupe 1986 [24] | D: 70% (nurses) use as a diagnostic device for testing the psychogenesis of complaints |
| Ernst 1997 [27] | D: 24% of nurses believed that placebo response differentiates organic and functional disease (32% no, 41% do not know)  P: 58% believed there is a responder personality (8% no, 29% do not know) |
| Berger 1999 [28] | D: 68% of interns considered placebo a useful test separating organic and functional disease; 48% were likely to use a placebo when they suspected factitious pain |
| Berthelot 2001 [29] | P: Patients believe that the placebo effect more common in elderly patients (83%), women (48%), younger patients (36%) or men (26%), in psychologically fragile persons (83%), hypochondriacs (60%), trusting in physicians (66%) or treatment (64%).  Nurses believe that the placebo effect more common in elderly patients (68%), women (62%), younger patients (36%) or men (32%), in psychologically fragile persons (62%), hypochondriacs (17%), trusting in physicians (73%) or treatment (81%) |
| Hrobjartsson 2003 [30] | D: 25% of GPs, 13% of hospital clinicians and 15% of private specialists reported placebo use to test whether the condition was functional or organic |
| Nitzan 2004 [31] | D: Among placebo users 37% (physicians) and 18% (nurses) had used them as diagnostic tool to separate organic and functional or simulated disease |
| Sherman 2007 [33] | D: Among placebo users 4% (physicians) had used them as diagnostic tool; 80% disagreed with the statement, “I think a placebo intervention can help distinguish symptoms that have a psychogenic versus an organic origin.” |
| Bernateck 2009 [35] | D: 26% (19% physicians, 29% nurses) have already used placebo application as a diagnostic tool |
| Chen 2009 [36] | D: 27% (patients) find it appropriate use a placebo to find out whether a pain is organic or not, further 24% would do so in rare occasions  P: 65% (patients) agree completely or mostly that effectiveness of a placebo is dependent on the personality of the patient (27% unsure, 8% disagree) |
| Fässler 2009 [37] | D: 21% of physicians used placebo to test whether pain is psychogenic or organic (15% of users plus non-users); 8% believed that pure placebos can be used to distinguish between psychogenic or organic pain (64% not, 28% do not know)*  P: 75% believed that there are persons with certain characteristics who rather respond to placebos (4% not, 21% do not know)* |

*unpublished information from author
