# Supplementary material for: Frequency and circumstances of placebo use in clinical practice - a systematic review of empirical studies
Source: BMC Med. 2010 Feb 23;8:15. doi: 10.1186/1741-7015-8-15 (PMC2837612; doi:10.1186/1741-7015-8-15)
Supplement: Additional file 4 — Summary of findings regarding beliefs on whether placebos can be used for diagnostic purposes (D) and personality features of responders (P). [file 1741-7015-8-15-S4.DOC]

**Additional file 4: Summary of findings regarding beliefs and experiences on effectiveness of placebo treatment**

| **First author year** | **Effectiveness of placebo treatment** |
| --- | --- |
| Goldberg 1979 [18] | N: Placebo was seen as providing either some or marked positive initial response in 81% of patients and as eventually effective in 68% of the cases |
| Goodwin 1979 [19] | Ph: the majority believed that 20% of patients respond to a placebo injection with adequate relief of pain the day after abdominal surgery  N: the majority believed that up to 5% of patients respond to a placebo injection with adequate relief of pain the day after abdominal surgery |
| Gray 1981 [20] | N+Ph: 15% believed that more than 30% of patients obtain relief of abdominal pain one day after abdominal surgery with a placebo, 59% less than 20% of patients, 26% no patient |
| Lange 1981 [21] | Ph+N+Ps: In 45% of placebo administrations their effect was judged to be satisfying, in 46% unsatisfying (9% unclear or unknown) |
| Ernst 1997 [27] | N: 28% believed that more than half of patients responds to placebos, 27% half of patients, 45% less than half of patients; 29% believed that placebos can change objectively measured variables, 40% subjectively measured variables |
| Berger 1999 [28] | 36% of interns “underestimated” placebo efficacy and 17% “overestimated” it (presumably authors consider placebo response rates from 30% to 50% which they cite in the introduction as the correct rate) |
| Berthelot 2001 [29] | Pt: believed that 32% of patients respond occasionally and 21% consistently to placebo therapy  N: believed that 42% of patients respond occasionally and 23% consistently to placebo therapy |
| Hrobjartsson 2003 [30] | Ph: 51% believed that placebo treatments have an effect on subjective symptoms, 32% on both subjective symptoms and objective signs, 9% no effect |
| Nitzan 2004 [31] | Ph+N: 33% of those who used placebo believed that placebo treatments are generally effective, 61% occasionally effective |
| Lim 2007 [32] | S: 33% believed that there is evidence that placebo treatments have beneficial effect on organic disease |
| Sherman 2007 [33] | Ph: 16% believed that placebo treatments are often effective, 58% sometimes effective, 21% rarely effective; depending on the condition participants expect after placebo administration psychological benefit only (26%-50%), physiological benefit only (0 to 2%), or both (20%-40%) |
| Bernateck 2009 [35] | 50% (29% Ph, 64% N) believed that placebos are always or often effective, 34% sometimes effective (44%, 39%), 16% not known (29%, 8%) |
| Chen 2009 [36] | Pt: 20% agree completely or mostly that placebos work in general (65% unsure, 15% disagree). 17% agree completely or mostly that a placebo can provide complete pain relief (47% unsure, 36% disagree); 21% agree completely or mostly that the effect of a placebo can last as long as that of the real treatment (56% unsure, 23% disagree) |
| Fässler 2009 [37] | Ph: 26% agreed that pure placebos must be rejected in principle because it is ineffective, 57% disagreed, 17% uncertain; 19% agreed that impure placebos must be rejected in principle because it is ineffective, 70% disagreed, 12% uncertain; 19% agreed that clinical effects of placebo interventions are mostly negligibly small, 61% not small, 20% uncertain |

Ph = physicians, Ps = psychologists, Pt = patients, N = nurses, S = students
