# Supplementary material for: Frequency and circumstances of placebo use in clinical practice - a systematic review of empirical studies
Source: BMC Med. 2010 Feb 23;8:15. doi: 10.1186/1741-7015-8-15 (PMC2837612; doi:10.1186/1741-7015-8-15)
Supplement: Additional file 5 — Summary of findings regarding beliefs and experiences on effectiveness of placebo treatment. [file 1741-7015-8-15-S5.DOC]

**Additional file 5: Summary of findings regarding ethical issues**

| **First author year** | **Ethics findings** |
| --- | --- |
| Shapiro 1973 [15,16] | Ph: Physicians were asked to rate agreement on a scale from 1 (completely agree) to 7 (completely disagree): conscious placebo use unethical 1.82, conscious placebo use inevitable 2.62, hostility underlies placebo use 2.64, inadequate MDs use placebos 2.36, deluded MDs deny placebo use 2.36; graduate psychoanalysts were extremely critical towards placebo use |
| Goldberg 1979 [18] | N: 34% believed placebo is rarely indicated, 4% never; none believed that it is often indicated. 19% believed that placebo use is acceptable for treatment, feelings associated with administering placebo (when described) were positive in 5%, neutral in 34%, somewhat negative in 18% and strongly negative in 21% |
| Gray 1981 [20] | N+Ph: “Over 60%” considered the observed placebo used as ethical, 25% unethical, 13% undecided |
| Lange 1981 [21] | Ph+N+Ps: Trend for a more favourable attitude towards placebo application with increasing professional experience |
| Thomson 1982 [22] | Ph: No direct questions on ethics but questions on behaviour in hypothetical situation: depending on the situation up to 84% would consider using a placebo intervention, in most situations described, however, about half of the physician would “definitively not” use a placebo |
| Classen 1985 [23] | Ph: 87% considered application of placebos (pure or impure?) justified (43% of those for scientific investigations, 52% for clinical trials and 63% for treatment purposes) |
| Saupe 1986 [24] | N: 30% feel a strong conflict because placebos imply “fooling the patient”, but 56% accept its use just for pragmatic reasons because it sometimes works, 3% wants to abolish placebo entirely |
| Lynöe 1993 [26] | Ph+Pt: See separate summary |
| Ernst 1997 [27] | N: 35% considered placebo use ethical in principle (51% not, 11% do not know), 73% believed that the use of placebo means deceiving the patient (17% not, 7% do not know) |
| Berger 1999 [28] | Interns with no prior knowledge of placebo use appeared more likely to exclude placebo use under any circumstances |
| Berthelot 2001 [29] | Pt: 45% thought physicians should use placebos, 27% thought physicians should tell if they do so, 28% would agree to take a placebo  N: 66% thought physicians should use placebos, 3% thought physicians should tell if they do so, 45% would agree to take a placebo |
| Hrobjartsson 2003 [30] | Ph: 46% considered placebo treatment ethically acceptable, 40% unethical, and 14% were uncertain. Of the respondents who considered placebo treatments unethical 50% reported that they still had prescribed them |
| Nitzan 2004 [31] | Ph+N: 5% thought placebo use should be categorically prohibited. 29% stated that placebos are permitted after notifying the patient that he or she is receiving a placebo |
| Lim 2007 [32] | S: 71% felt that placebo is an acceptable form of therapy; 13% considered placebo prescription ethical also in organic disease with known definitive treatment, 64% for organic disease with no treatment, and 82% for purely psychogenic disease |
| Sherman 2007 [33] | Ph: 12% said that placebo use should be categorically prohibited, 46% considered it acceptable in cases where research supported its efficacy, 31% if benefit was anticipated, 21% if the patient was informed about placebo use, or 9% if experience of colleagues supported it |
| Tilburt 2008 [34] | Ph: 3% considered it obligatory to recommend treatment primarily to promote patients’ expectations, 59% permissible, 31% permissible only in rare circumstances and 7% never permissible |
| Chen 2009 [36] | Pt: No direct questions on ethics but patients were asked whether they would consider placebo application appropriate in ten different hypothetical situations: Between 19% and 50% would consider it appropriate “most of the time” or “definitely” if given for the benefit of the patient while most considered it inappropriate if given for the benefit of physician (e.g. in difficult patients or for meeting prescription expectations in a new patient) |
| Fässler 2009 [37] | Ph: 45% agreed that placebo use must be rejected because it implies deception, 17% were uncertain, 37% disagreed; 55% considered it acceptable for the benefit of the patient and for minimizing harm, 20% were uncertain and 26% disagreed |

Ph = physicians, Ps = psychologists, Pt = patients, N = nurses, S = students
