# Supplementary material for: Frequency and circumstances of placebo use in clinical practice - a systematic review of empirical studies
Source: BMC Med. 2010 Feb 23;8:15. doi: 10.1186/1741-7015-8-15 (PMC2837612; doi:10.1186/1741-7015-8-15)
Supplement: Additional file 6 — Summary of the study by Lynöe et al. [26]. [file 1741-7015-8-15-S6.DOC]

**Additional file 6: Summary of the study by Lynöe et al. [26]**

| **Case 1** | Self-employed 44 year old male with a cold demanding an antibiotic – the physicians prescribes it after the patient insists.   - 34% of patients and 8% or physicians found this acceptable as the physician ought to oblige the patient’s wish - 42% vs. 14% found it acceptable as the placebo effect will be high - 59% vs. 82% found it unacceptable because the treatment goes against science - 48% vs. 11% completely disagreed to the statement that the procedure is unacceptable due to possible side effects and that the physician should have ordered a preparation without side effects, e.g. a pure placebo |
| --- | --- |
| **Case 2** | 42 year old woman with newly diagnosed advanced cancer for which a curative treatment is not possible, but the patient still has great hopes of being cured by treatment.   - 63% of patients and 9% of physicians found the procedure acceptable as the risk is small that the patient discovers that she receives placebo - 69% vs. 18% found the procedure acceptable as it preserves the patient’s hope without making her final time unbearable - 43% vs. 85% found the procedure unacceptable as the patient has not been given adequate information - 50% vs. 69% found the procedure unacceptable because, should it be common knowledge, faith in the system might be shaken |
| **Case 3** | 42 year old man with newly diagnosed leukaemia for which effective treatment is available. The patient refuses treatment (unnatural, side effects unacceptable) and asks the physician to receive mistletoe treatment which the physician considers to be no more than a placebo.   - 64% of patients and 81% of physicians agreed that the physician ought to use any means to persuade the patient to undergo ordinary treatment - 67% vs. 79% agreed that the physician ought to respect the patient’s desire to refuse ordinary treatment - 72% vs. 41% agreed that the physician should apply mistletoe in order to not reduce the placebo effect - 20% vs. 51% agreed that the physician should not agree to the patient’s desire for mistletoe since it goes against science |
